# Zinc and hydroxyapatite co-localize during in vitro *E. coli* biofilms mineralization

**DOI:** 10.1038/s41598-025-33942-3

**Published:** 2026-01-14

**Authors:** Leona J. Bauer, Laura Zorzetto, Ernesto Scoppola, Yannick Wagener, Ioanna Mantouvalou, Cécile M. Bidan

**Affiliations:** 1 Institute for Physics and Astronomy, Technische Universität Berlin, Hardenbergstrasse 36, 10623 Berlin, Germany; 2 Department of Biomaterials, Max Planck Institute of Colloids and Interfaces, 14476 Potsdam, Germany; 3 SyncLab, Helmholtz-Zentrum Berlin für Materialien und Energie, Albert-Einstein-Str. 15, 12489 Berlin, Germany

**Keywords:** *E. coli* biofilm, Mineralization, (confocal) micro-XRF, WAXS, Zn accumulation, Hydroxyapatite, Biotechnology, Materials science, Microbiology

## Abstract

Biofilms are biological materials that form as bacteria protect themselves from environmental challenges secreting extracellular matrix and accumulating minerals under specific conditions. To understand biofilm formation and mineralization, we grew *Escherichia coli* on agar plates containing a nutritive and mineralizing medium. Previous studies showed that the alkaline phosphatase (ALP) present in *E. coli* biofilms leads to hydroxyapatite precipitation in such conditions. Here, we introduced X-ray fluorescence techniques as powerful tools to analyze the composition of mineralized biofilms in two and three dimensions. In addition to calcium and phosphate, we found that the traces of zinc introduced *via* the nutrients and bacteria, also accumulates in the mineralized regions. Wide-angle X-ray scattering performed on dry mineralized biofilm powder also revealed a shortening of the hydroxyapatite crystal lattice, which could indicate partial substitution of calcium by zinc. Using an abiotic model, we reproduced enzymatic hydroxyapatite mineralization with pure ALP, without bacteria nor extracellular matrix. In this model, hydroxyapatite distortion increased with zinc concentration, whereas crystal size decreased. Since zinc is a ubiquitous element that is essential to bacteria metabolism but also antibacterial, these results are fundamental for biofilm research and call for further studies on zinc management in biofilms.

**Supplementary Information:**

The online version contains supplementary material available at 10.1038/s41598-025-33942-3.

## Introduction

 Biofilms are microbial tissues made of bacteria embedded in a self-produced extracellular matrix. This organic matrix consists of a mesh of proteins and polysaccharides^1^. In specific conditions, the microenvironment created by the bacteria leads to mineral precipitation within the biofilm. Such phenomenon is of interest not only to understand biomineralization in geomicrobiological contexts^2^, but also to anticipate bacteria induced tissue calcification leading to serious pathologies such as kidney stones or as dental calculus leading to periodontitis^3^. Our recent study performed on a model system showed that *Escherichia coli (E. coli)* biofilms grown in the presence of calcium chloride and organic phosphate (β-glycerophosphate) undergo mineralization and the calcium-phosphate crystals formed are hydroxyapatite^4^. That study focused on identifying the mineral phase, its spatial distribution in the biofilm, and the role of the bacterial enzyme alkaline phosphatase (ALP) in the mineralization process^5^, without examining the influence of additional components beyond calcium and organic phosphate. However, the potential role of chemical trace elements other than calcium and phosphate in the process of biofilm mineralization remained understudied and exploring this role requires powerful and multimodal analytical approaches.

Zinc is omnipresent in biological materials produced by multiple organisms. It fulfils diverse essential roles for the cells and has various effects depending on its form (bound or free ion)^6^. For example, Zn is important for the metabolism, proliferation and signaling of many cell types^7,8^. In bacteria cytoplasm, free zinc ions are in very low concentration but larger amounts of zinc bind to proteins, especially to amino acids like cysteine^9,10^. Zn was also shown to favor cell-cell adhesion *via* adsorption on surface proteins^11^. Zinc also plays a fundamental role in the early stage of osteogenesis, where Zn-apatite offers the first nucleation site for the hexagonal crystal to grow^12^. Zinc can contribute to enhanced properties of biological materials, as demonstrated in sharp organismal tools from various animals (e.g., teeth and claws), where zinc atoms are proposed to bind nitrogen atoms of extracellular proteins shaping the tissue^13^. It was also shown to be incorporated into calcium-phosphate minerals formed *via* enzymatic mineralization within hydrogels for tissue engineering application^14^. In such cases, Zn^2+^ ions substitute Ca^2+^ ions in the crystal lattice and the resulting Zn-doped hydroxyapatite enhances both antibacterial activity and bone cell viability on the hydrogel. Indeed, zinc is also a well-known antimicrobial agent and this property is widely used in biomaterials, where the incorporation of zinc aims at avoiding the adhesion of bacteria and further development of biofilms^15,16^. However, some studies show that high concentrations of zinc can also promote pneumococci and staphylococci aggregation, as well as biofilm formation^17,18^. Finally, zinc has high affinity for *E. coli* ALP^19^, thereby mediating the activity of this enzyme and inducing potential impacts on downstream mechanisms at larger scales^20^.

To understand the role of Zinc, the localization within the biological system is necessary. Different imaging techniques such as fluorescence microscopy^16,17^, SEM-EDX^21^ and ICP-MS^22^ can be used which are typically applied to cross sections due to their surface sensitivity.

Here, we utilize X-ray fluorescence spectroscopy (XRF) techniques^23^ which offer non-destructive imaging in combination with the possibility to probe beneath the surface. 2D imaging is facilitated by using focused X-rays from X-ray tubes or synchrotron radiation sources to locally excite the atoms of a sample and detect the emitted element-specific characteristic radiation. This micro-XRF (MXRF) technique requires little to no sample preparation and when used under pre-vacuum atmosphere, allows imaging of a wide range of elements (Na-U). When using a second optic in the detection channel in a confocal arrangement, a probing volume is created, enabling depth-sensitive measurements^24^. Confocal MXRF (CMXRF) thus renders 3D elemental imaging of a sample feasible^25^ with size limitations in the range of millimeters.

As XRF techniques are useful to create elemental maps, wide-angle X-ray scattering (WAXS) enables researchers to retrieve a range of information on crystalline materials^26^. Analyzing Bragg peaks, it is possible to derive the crystal dimensions and identify mineral phases by determining parameters of the crystal lattice, e.g. the dimensions of the crystal unit cells along three dimensional axes (a-, b- and c- parameters corresponding to the a, b, c axes, respectively), estimated through the distance between parallel planes of atoms within a crystal structure (d-spacing)^27^.

Combining X-ray fluorescence and wide-angle X-ray scattering, this work presents a detailed analysis of mineralized *E. coli* biofilms grown on nutritive agar substrates at 28 °C, to assess the potential role of trace elements in the process of biofilm mineralization. MXRF, and CMXRF were used to identify and locate key elements such as Ca, P and Zn within intact mineralized biofilms grown for different durations before fixation (5, 7 and 10 days) and in the presence of an organic phosphate source (β-glycerophosphate) and two different Ca concentrations.

Zn appeared to accumulate in parts of the mineralized region together with Ca and P. A semi-quantitative elemental analysis of the nutrients and bacteria used to grow the biofilms, showed that Zn is intrinsically present in substantial amounts in all the ingredients of the growth medium, except in the agar itself. To study the influence of trace elements on the nanostructure of the hydroxyapatite crystals, WAXS was then performed both on mineralized biofilm powder and on minerals from an additional abiotic model reproducing ALP-mediated mineralization of hydroxyapatite (without bacteria nor extracellular matrix) and where Zn concentration could be varied without interfering with bacteria metabolism. A distortion of the hydroxyapatite lattice was measured in all materials, depended on amount of Zn, and was compatible with possible substitutions of Ca atoms by Zn atoms in the crystal lattice.

## Materials and methods

### Biofilm growth and mineralization

Biofilms were grown from the strain *E. coli* K-12 W3110 and mineralized following the approach detailed in^4^. We report here the methods used both in the previous and current work. Salt-free LB agar (Luria/Miller) plates (control medium) were prepared with 1.8% w/v agar (Roth 2266), 1% w/v tryptone (Roth 8952), and 0.5% w/v yeast extract (Roth 2363). For the mineralizing media, CaCl_2_ (Sigma-Aldrich 223506) and sodium ß-glycerophosphate (Sigma-Aldrich 35675) solutions were sterile-filtered and added to the autoclaved (still liquid) salt-free LB agar to reach a final concentration of 10 mM ß-glycerophosphate and 1 or 10 mM CaCl_2_. These conditions were, respectively, named Ca1P10 and Ca10P10. The 10 mM β-glycerophosphate concentration and 1 mM calcium concentrations were chosen according to values reported in literature for phosphate and calcium ions in human saliva^28^. For calcium, 10 mM concentration was also considered, as it was proven to lead to biofilm mineralization both in liquid and solid medium^4,5^. Both calcium concentrations produce mineralizing biofilms, but the latter leads to faster mineralization which is more desirable for an in vitro model. We deliberately did not add extra Zn to the agar medium for biofilm growth, because ZnCl_2_ can interfere with agar solidification process. However, Zn was intrinsically present through the nutrients (tryptone and yeast powder) and its concentration in the solid growth medium was quantified using micro-XRF methods.

In each plate, biofilms were inoculated with 5 µL droplets of bacterial suspension (OD600 ∼5.0) obtained after overnight culture of a single *E. coli* microcolony in 5 mL LB medium (Luria/Miller) (Roth X968) at 37 °C and 250 rpm. After seeding, the droplets were left to dry and the agar dishes were incubated at 28 °C for 5, 7 and 10 days.

### Biofilm preparation

**Fig. 1:**
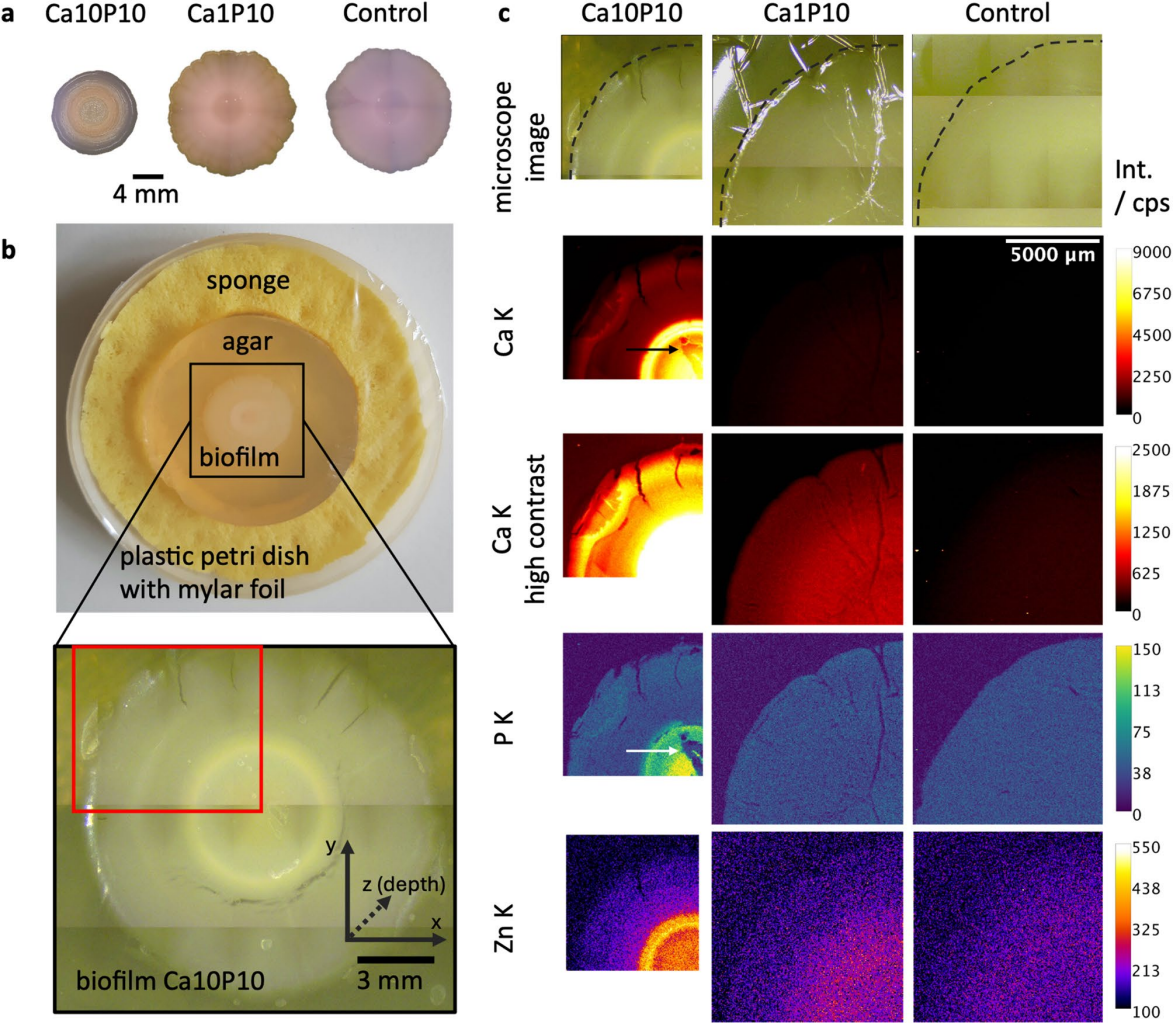
Elemental distributions of MXRF measurements on three biofilms grown for 10 days under different mineralization conditions (Ca10P10, Ca1P10, Control). **a** shows microscopy images of a biofilm of each group. **b** shows a photograph and a microscope image of a Ca10P10 biofilm in its sample holder. The red rectangle in the lower images marks the area measured by MXRF. **c** shows the Ca K, P K and Zn K distribution of the three groups and an optical image of the measured area. The black dotted line goes along the outer rim of the biofilms. The black and white arrow mark regions where Ca and P K signal is absorbed by water droplets or surface topography. The color scales show the X-ray fluorescence net peak intensities in counts per second (cps). The length scale is the same for all three measurements. More elements are shown in Supplementary Fig. S2.

All biofilms were fixed upon 2 h contact with 4% paraformaldehyde (PFA, Bioster Ar106) in phosphate-buffered saline (PBS, Sigma-Aldrich P4417). The PFA excess was then removed and the biofilms were rinsed with PBS. For MXRF and CMXRF measurements the biofilms on agar substrate were placed in flat plastic cups, surrounded by a wet sponge and covered with a 1.4 μm thick mylar foil (see Fig. 1b). The foil was attached to the cup with tape and sealed with an elastic parafilm stripe.

### Pellet preparation

Using powder of the single components (agar, tryptone, yeast, *E. coli*) as well as from biofilms (Ca10P10 and control), pellets were prepared. Additionally, a pellet of hydroxyapatite (HAp) powder was prepared as a reference sample. The density of the pellets was derived from measuring the weight and the thickness of the pellets. The radius of the pellets was the same for all pellets and defined by the used press (1.3 cm), except for the *E. coli* pellet which was pressed with a spacer ring to produce a smaller radius due to a small amount of powder.

### Mineralization from purified ALP - abiotic model

The abiotic model simplifies the experimental conditions to the sole ingredients necessary to the enzymatic mineralization process of HAp in solution. Alkaline phosphatase (ALP, 100 U) from *E. coli* (Sigma Aldrich P5931, lot# 039M4019V) was dissolved in glycine buffer (0.1 M glycine/NaOH pH 10.4, 1 mM MgCl_2_, 1 mM ZnCl_2_) to reach the final concentration of 20 U/mL. The enzymatic solution (200 µL) was mixed with other water-based solutions with concentrations of CaCl_2_ and sodium ß-glycerophosphate equal to the Ca10P10 agar (0.1 M) and concentrations of either ZnCl_2_ or SrCl_2_ ranging from 0.1 mM to 2 mM (Table 1). The same solutions without ALP were used for control experiments. All solutions were incubated overnight at 28 °C. We observed the formation of a precipitate after the incubation period only in the solutions containing ALP.

**Table 1:** Summary of the calibration curve samples. To reach the final volume of 10 mL, 1 mL of ß–glycerophosphate (0.1 M) and 1 mL of CaCl_2_ (0.1 M) were added to each sample. Additionally, 0.2 mL of the solution containing the ALP enzyme dissolved in a glycine buffer (20 units/mL) were added.

Water volume (mL)	0.1 M ZnCl_2_ volume (µL)	0.1 M SrCl_2_ volume (µL)	Label
7.80	–	–	Zn – traces
7.79	10	–	Zn – 0.1 mM
7.78	20	–	Zn – 0.2 mM
7.75	50	–	Zn – 0.5 mM
7.70	100	–	Zn – 1.0 mM
7.60	200	–	Zn – 2.0 mM
7.79	–	10	Sr – 0.1 mM
7.78	–	20	Sr – 0.2 mM
7.75	–	50	Sr – 0.5 mM
7.70	–	100	Sr – 1.0 mM
7.60	–	200	Sr – 2.0 mM

### (Confocal) micro-X-ray fluorescence measurements

**Fig. 2:**
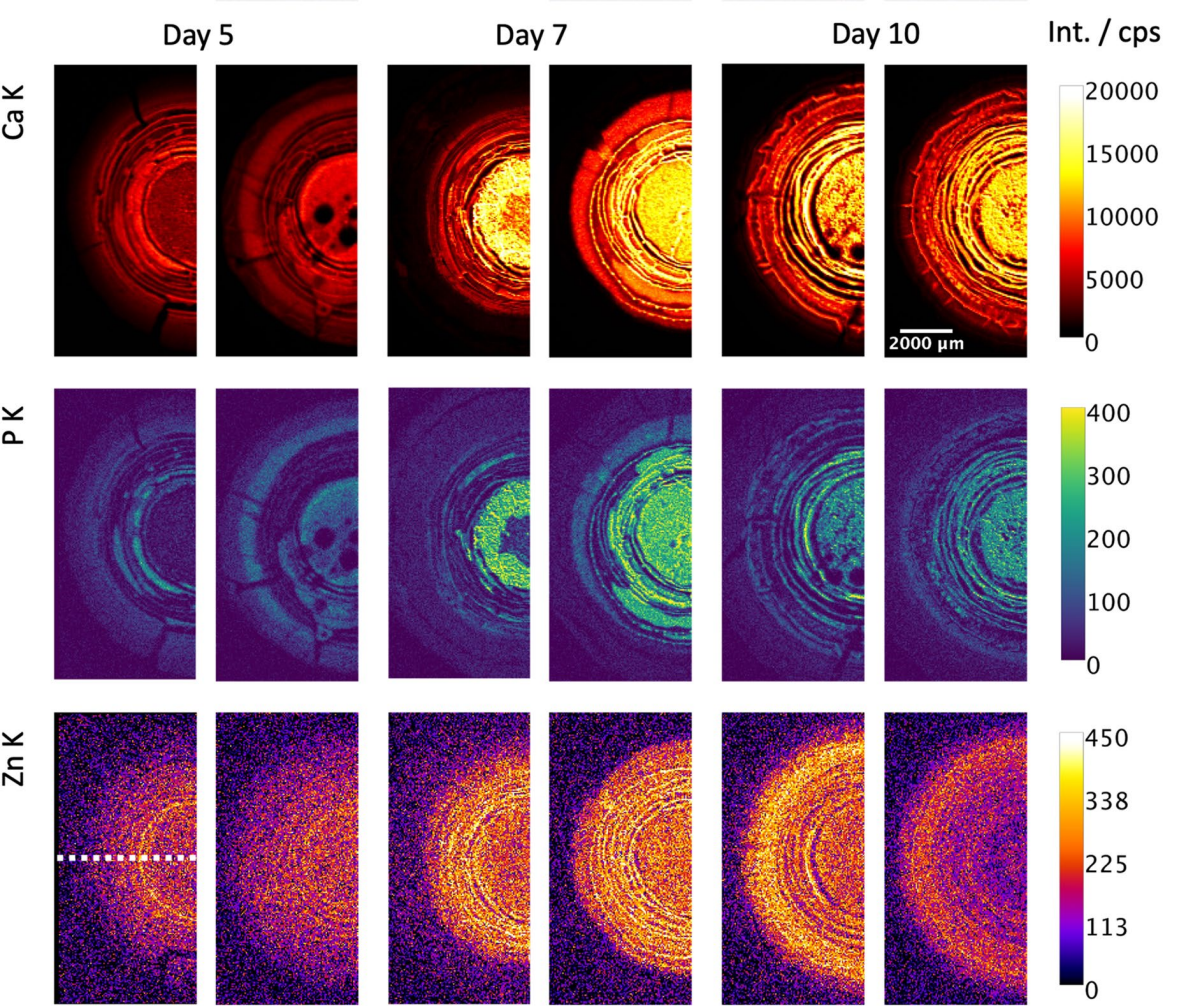
Elemental distribution in Ca10P10 biofilms. MXRF elemental distributions of Ca, Zn, and P measured on biofilms grown for 5, 7 and 10 days on the mineralizing medium Ca10P10. The color scales show the X-ray fluorescence intensities in cps.

**Fig. 3:**
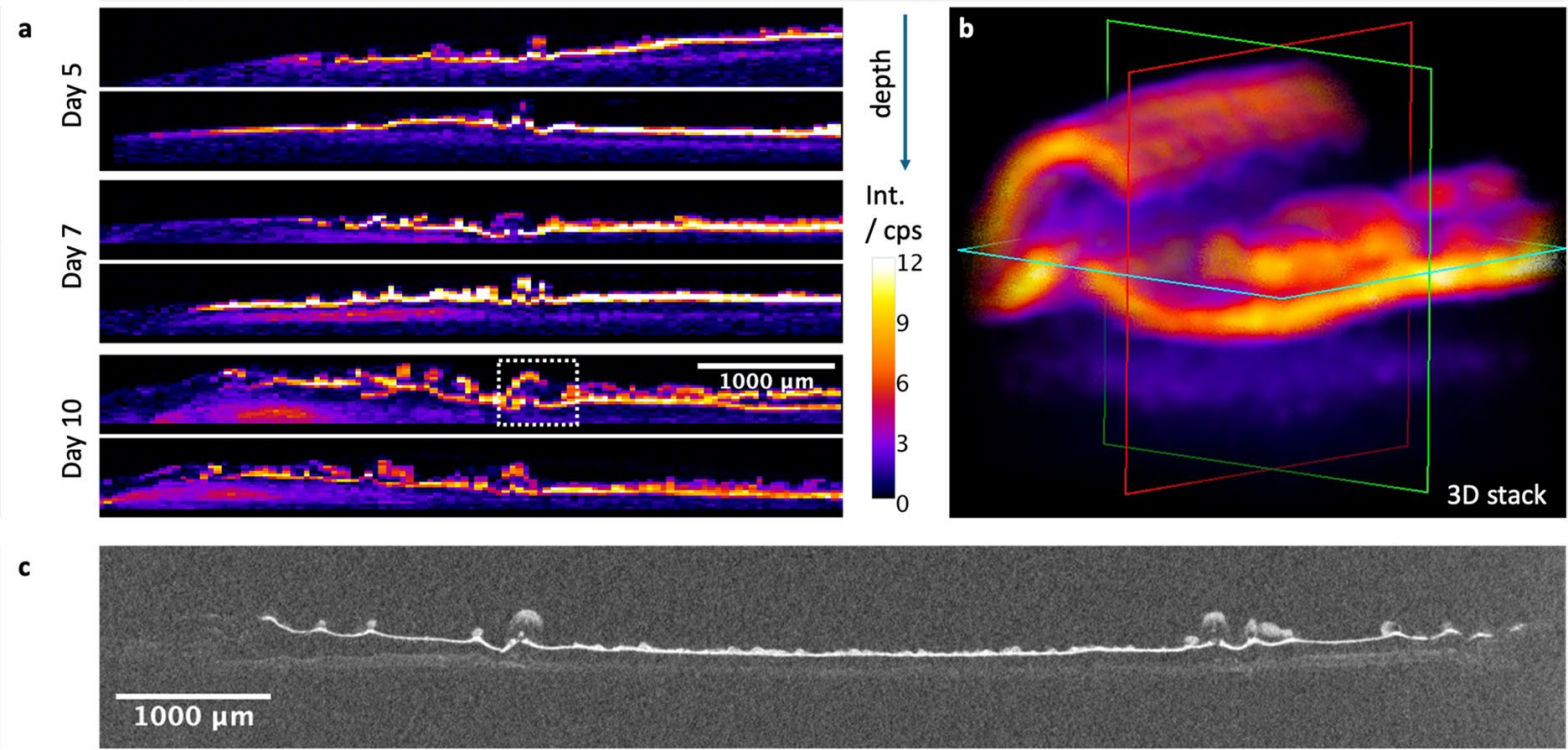
Mineral distribution in Ca10P10 biofilm thickness. **a** Zn K distribution of virtual slices into the depth for all biofilms in Fig. 2 along the white dotted line measured by CMXRF. **b** 3D Zn K distribution of a detail of a biofilm grown for 10 days (marked with the white rectangle). ** c** Microtomography of a biofilm cross section. Biofilms were grown on mineralizing medium (Ca10P10), fixed and stabilized by pouring another agar layer on top of the biofilm prior to the measurement.

X-ray fluorescence measurements were performed with a commercial spectrometer (Bruker M4 Tornado Plus) using an Rh microfocus X-ray tube with 50 kV and 1 mA excitation^29^ and a polycapillary optic for focusing of the radiation. The spectrometer is equipped with two energy-dispersive detectors, one for MXRF mapping and one with an additional polycapillary optic for CMXRF measurements. Using MXRF spectroscopy 2D maps of the biofilms, pellets and material from the abiotic system were measured. Using CMXRF spectroscopy virtual 2D slices and a 3D volume of the biofilms were obtained. Virtual slices are measured without sectioning by measuring along the x and z axis (Fig. 1b) into the depth. This is possible due to the depth sensitivity of the CMXRF technique. The XRF spectra were deconvolved using the Esprit Software of the spectrometer (Fig. 2) and an in-house software SpecFit^29^ (Figs. 1 and 3). The resulting net peak intensities in cps for certain fluorescence lines were visualized using ImageJ 2.1.0/1.53s.

X-rays are absorbed in matter following an exponential law with the absorption depending on the energy of the radiation and the composition/density of the material. This self-absorption limits the depth inside the sample from which elemental information can be derived. The so called “information depth” is defined as the thickness of a material where 63% (1/e) of the measured fluorescence is generated. Information depth values for different samples and elements of interest range from a few micrometers to millimeters^30^. In case of the biofilm samples presented here this means, that the main information of P is derived from around 20 μm, Ca is derived from around 80 μm, while the information on Zn is derived from around 570 μm. The values were calculated based on the composition and density of the pellet pressed from mineralized biofilm material (Table 2).

Quantification was performed on averaged sum spectra measured on the pellets (see Supplementary Fig. S9) using the proprietary evaluation package FPQ-Tools, developed by Bruker. For this purpose, the density and thickness of the pellets must be given, as the pellets are not infinitely thick for hard X-rays. Additionally, there are elements (i.e. C, O, H) contained in the pellets that cannot be measured by MXRF due to the properties of the used optics and the absorption on the way to the detector. As these elements cannot be measured, but are part of the matrix of the samples, this part is called the ‘dark matrix’. To be able to quantify the composition of the elements that can be measured, assumptions on the dark matrix have to be made. The assumptions we used for the dark matrix of all pellets are detailed in the Supplementary Table S1.

**Table 2:** Measurement parameters for the MXRF and CMXRF measurements.

Figure	Panel	Measurement mode	Step size	Measurement time per spectrum (lifetime)
1c	Left column (Ca10P10)	MXRF	50 μm x 50 μm	1.75 s
1c	Center and right column (Ca1P10 and control)	MXRF	50 μm x 50 μm	0.85 s
2	All	MXRF	50 μm x 50 μm	0.30 s
3a	All	CMXRF, virtual 2D slices	50 μm x 20 μm width x depth	51 s
3b		CMXRF, 3D volume	20 μm x 20 μm x 20 μm	18.5 s

### Micro computed tomography

After 10 days of growth, a Ca10P10 biofilm was fixed and covered with a layer of liquefied agar-agar (Roth 2266) poured on top of it. After the agar-agar solidification, the biofilm was cut out from the cultivation dishes using the upper part of disposable plastic Pasteur pipettes (12 mm diameter). The sample was kept sealed to avoid water evaporation and was characterized using an X-ray microtomography scanner (RX Solutions EasyTom160/150 tomographic unit) with a voxel size of 8 μm at 70 kV and 130 µA.

### Wide angle X-ray scattering

Lyophilized biofilms and mineral precipitate obtained in the abiotic system were characterized by WAXS. The lyophilized biofilms were filled into a 2 mm Teflon sample holder with multiple holes of 5 mm diameter. The samples were overlaid with a Kapton film on both sides. WAXS measurements were performed at the µSpot beamline at synchrotron facility BESSY II operated by the Helmholtz-Zentrum Berlin^31^. For the abiotic precipitates, the majority of the supernatant was removed and 20 µL of each of the hydrated precipitates were deposed on a layer of Kapton tape in a sample holder and let dry overnight under vacuum (see Supplementary Fig. S1a). The measurements were carried out using a B4C/Mo Multilayer monochromator (2 nm period), with an X-ray energy of 15 keV for the biofilm samples and 18 keV for the mineral precipitate. A sequence of pinholes was used to select a 100 × 100 µm^2^ spot size. Data were normalized to the primary beam intensity and the background was subtracted. Transmission through the sample was calculated from an X-ray fluorescence signal collected from a lead beam stop using a RAYSPEC Sirius SD-E65133-BE-INC detector. The detector was equipped with an 8 μm beryllium window, where the primary beam intensity was monitored using an ion chamber. Scattering data were collected with an Eiger 9 M detector with 75 × 75 µm^2^ pixel area. For each sample, four spots were measured and diffraction patterns were collected with an exposure time of 15 s. Further data processing and reduction was performed using the directly programmable data analysis kit (DPDAK)^27^. Diffraction patterns were radially integrated and the scattered intensity $\:I\left(q\right)$ was calculated as a function of the momentum transfer $\:q$, defined as:

1 $$\:q=\frac{4\pi\:}{\lambda\:}\mathrm{s}\mathrm{i}\mathrm{n}\left(\frac{2\theta\:}{2}\right)$$

with $\:\lambda\:$ and 2$\:\theta\:$ the photon wavelength and the scattering angle, respectively. The sample-to-detector distance was set to approximately 330 mm, yielding q-ranges of 0.1–30 nm^− 1^ at 15 keV and 0.1–42 nm^− 1^ at 18 keV, and was calibrated using quartz powder. Data were analyzed with an in-house Python based software (Python 3.8.8). From the reduced data, we focused on the 002 peak of hydroxyapatite, which allowed us to calculate both the crystallite size along the c-axis and the c-parameter that is the interplanar space along the crystal c-axis. For a schematic explanation of these parameters, see Fig. S1b. We estimated them using the following equations:

2 $$\:crystallite\:size\:\left(nm\right)=\:\frac{K\lambda\:}{\beta\:\mathrm{c}\mathrm{o}\mathrm{s}\left(\theta\:\right)}$$

3 $$\:c-parameter=\:\frac{4\pi\:}{{q}_{002}}$$

with $\:K$ shape factor equal to 0.9, $\:\beta\:$ equal to the full width of half maximum in radians and $\:\theta\:$ the Bragg angle of the 002 peak.

## Results

To detail our understanding of the chemistry of mineralized *E. coli* biofilms, we first grew the bacteria on nutritious medium (control medium) or on mineralizing medium, i.e. control medium supplemented with an organic phosphate source and calcium ions. Two calcium concentrations were tested in this work, namely 1 mM (Ca1P10) and 10 mM (Ca10P10). Biofilms grown in mineralizing conditions presented a whitish ring-shaped area in their center (Fig. 1a, b) that presents a scattering pattern compatible with hydroxyapatite minerals (Fig. S1c), in accordance with a previous study^4^.

### Distribution of elements in mineralized *E. coli* biofilms

A spatially resolved elemental analysis was first conducted using MXRF. For this purpose, the biofilms were fixed with 4% PFA and remained on the agar during the measurement. Figure 1c shows representative results obtained on quarters of biofilms grown in the different mineralizing conditions (Ca10P10, Ca1P10, Control). While the distributions of the elements Ca, P and Zn are the most relevant for this study, more elements are shown in Supplementary Fig. S2. All three elements show distinct distributions in the biofilms as a function of the growth media used. Indeed, no or very low signal is detected across the biofilm in control and mild mineralizing conditions (Ca1P10), whereas on stronger mineralizing medium (Ca10P10), the three elements are clearly concentrated in the center of the biofilm and seem to co-localize with the mineralized region. Additional averaged XRF spectra for the different regions of the biofilm with strong mineralizing medium are shown in the Supplementary Fig. S3. These spectra show that P, Ca and Zn show significantly higher intensity in the center and in a ring-like structure around the center than in the rest of the biofilm. While the P intensity, which originates from the surface, is slightly decreased in the ring area, Ca and Zn show a higher intensity, which could be due to a higher thickness of the ring structure (see the additional virtual slices in Supplementary Fig. S4). While the hydroxyapatite nature of the minerals explains such distribution for the Ca and P elements, the concentration of Zn in this area was not expected. The differences in the distribution of Ca and P and the distribution of Zn can be explained by the different fluorescence energy of the K X-ray radiation. The K fluorescence energy for light elements (Ca, P) is lower than for Zn, resulting in a higher absorption in matter. In the areas where water droplets are present between the biofilm and the foil, the Ca K und P K fluorescence radiation is thus clearly attenuated, see the arrows in Fig. 1c), whereas the Zn K fluorescence is not significantly affected.

### Local co-accumulation of Ca, P and Zn in layers

To explore the origins of this co-localization, MXRF and CMXRF measurements were performed on biofilms grown for 5, 7 and 10 days on the mineralizing medium Ca10P10 and fixed prior to measurement. For each group, two biofilms grown in same conditions were measured. Figure 2 shows the Ca K, P K and Zn K distributions derived from MXRF measurements of half of the biofilms. After 5 days of growth, the distribution of Zn appears rather diffuse across the biofilm compared to the distributions of Ca and P, which show more defined and partially co-localized distribution patterns. Note that this time point corresponds to the onset of mineralization in these conditions^4^. After 7 days of growth, the accumulation of Ca and P is more pronounced in the central region of the biofilm. Zn is relatively homogeneously distributed in the region of Ca accumulation, which is slightly larger than the one of P accumulation. For better visualization we added radial profiles of the elemental distributions showing the co-localization of Ca and P in the Supplementary (Fig. S5). The deviations for Zn arise due to the different information depths of these elements. After 10 days of growth, the distributions are similar to the previous time point. Although the P signal appears to be slightly weaker than at day 7, this is not statistically relevant and the Ca/P and Zn/(Zn + Ca) ratios are the same for all growth times (see Supplementary Fig. S10).

With CMXRF, virtual slices (x- and z-axis, Fig. 1b) into the depth of the biofilms were measured along the white dotted line in Fig. 2 (Fig. 3a). While P and Ca can be measured only from the surface of the biofilm due to the information depth, Zn information can be derived from more than 500 μm. Zn seems to be distributed into two to layers that are following the complex morphology of the biofilms (Fig. 3). In the Supplementary Fig. S7 we show the distribution of Ca into the depth overlaid on the Zn K distribution. There, the different information depths and the co-localization of Ca and Zn in the first layer are visible. Interestingly, such structured layer is reminiscent to the hydroxyapatite mineralization pattern identified in previous study^4^ and by X-ray micro computed tomography, see Fig. 3c. While the layered structure is already visible in the biofilms grown for 5 days (~ 20 to 40 μm mineralized layer thickness), the separation of two layers (each ~ 40 to 60 μm thick) seems to become clearer after 7 and 10 growing days. Depth sensitive measurements on the biofilms grown under different mineralizing conditions also show a layered structure only for the strongest mineralizing medium (Ca10P10, see Supplementary Fig. S4). Furthermore, a Zn accumulation below the biofilm in the agar substrate becomes visible after 7 or 10 days (Fig. 3). This was further investigated by MXRF measurements on the biofilm and agar substrate separately after removing the biofilm (see Supplementary Fig. S8). Indeed, the accumulation in the agar shows enhanced intensities for all three elements (P, Ca, Zn). A detail of the layered structure marked with a white dotted rectangle was measured in 3D by CMXRF spectroscopy and shows a wave-like layered structure (Fig. 3b).

### Quantification in the individual biofilm elements

In order to assess the availability of Zn, Ca and P in the nutrients, pressed pellets of the powders of agar, yeast, tryptone and fixed and freeze-dried *E. coli* liquid culture were prepared. Additionally, two pellets were prepared from fixed, freeze-dried and ground *E. coli* biofilms, one grown for 10 days in mineralizing (Ca10P10) and one in control condition. The sum spectra obtained from the measurements on the pellets are shown in Supplementary Fig. S9. In Table 3, the mass fractions are quantified. Most of the matrix of the samples is composed of C, O, N and H, which cannot be measured with the MXRF spectrometer. Therefore, a good guess of this dark matrix is needed, see methods section. The main uncertainties in the quantification are due to these assumptions, resulting in semi-quantitative absolute concentration values. Nevertheless, relative differences in the fluorescence peak intensities are mirrored in the concentration values and can therefore be used for the interpretation of the results.

Agar shows the least amount of fluorescence elements, with only the S and Na concentration exceeding 0.1%. Yeast contributes predominantly with P, S and K, tryptone with Na, P, S, Cl and K and *E. coli* with Na, P, S and Cl. Except agar, all pellets show traces of Zn between 60 and 130 ppm. Both biofilms (Ca10P10 and control) show enhanced levels of Ca, with a significant increase in the mineralized biofilm sample (Ca10P10). The intensity of the Zn K fluorescence does not differ significantly between the two biofilm pellets (see Supplementary Fig. S9). Due to the different dark matrix contribution (87% vs. 95%), which has a significant influence on the mass fraction values for traces of high-Z atomic number, differences in the lower ppm range are difficult to interpret. The bigger differences are found in the distribution and accumulation of Zn rather than in their amount (see Fig. 1c).

**Table 3:** Mass fractions of quantified elements (bdl: below detection limit), in some pellets traces of Sr could be present. The main uncertainty for the quantification Lies in the assumption of the dark matrix, which yields semi-quantitative results.

Material	dark matrix	density/ g/cm³; thickness/ cm	Na	Mg	Si	*P*	S	Cl	K	Ca	Mn	Fe	Zn	Br	Rb
**agar**	99.25% C_12_H_18_O	0.9 0.148	0.38%	bdl	430 ppm	45 ppm	0.25%	490 ppm	30 ppm	110 ppm	bdl	50 ppm	bdl	40 ppm	bdl
**yeast**	52% CH_158_N_28_O_30_ 40% C_6_H_12_O_6_	1.29 0.105	35 ppm	bdl	600 ppm	1.4%	0.57%	460 ppm	5.9%	70 ppm	bdl	50 ppm	130 ppm	bdl	30 ppm
**tryptone**	94.5% C_11_H_12_N_2_O_2_	1.27 0.145	2.3%	90 ppm	650 ppm	0.77%	0.69%	0.46%	1.2%	420 ppm	bdl	30 ppm	80 ppm	bdl	bdl
**E. coli**	95% CH_158_N_28_O_30_	0.85 0.043	2.2%	bdl	0.13%	1.3%	0.38%	0.89%	730 ppm	150 ppm	13 ppm	180 ppm	60 ppm	bdl	bdl
**biofilm** **Ca10P10**	87% C_12_H_170_N_30_O_32_	1.01 0.07	1.7%	590 ppm	0.19%	4.0%	0.53%	0.81%	0.24%	5.4%	30 ppm	150 ppm	70 ppm	bdl	bdl
**biofilm control**	95% C_12_H_170_N_30_O_32_	1.1 0.04	1.4%	882 ppm	890 ppm	1.8%	0.54%	0.69%	0.34%	480 ppm	26 ppm	120 ppm	40 ppm	bdl	bdl

### Hydroxyapatite crystalline structure in presence of Zn

MXRF and CMXRF measurements show that Zn contained in the nutritive components of the substrates tends to be accumulated in the mineralized regions of the biofilm (Figs. 2 and 3). This could indicate that Zn is incorporated within the hydroxyapatite mineral as a doping element that would partially substitute Ca, which could be supported by the co-localization of calcium and zinc in the mineralized biofilm areas shown by the X-ray fluorescence data.

**Fig. 4:**
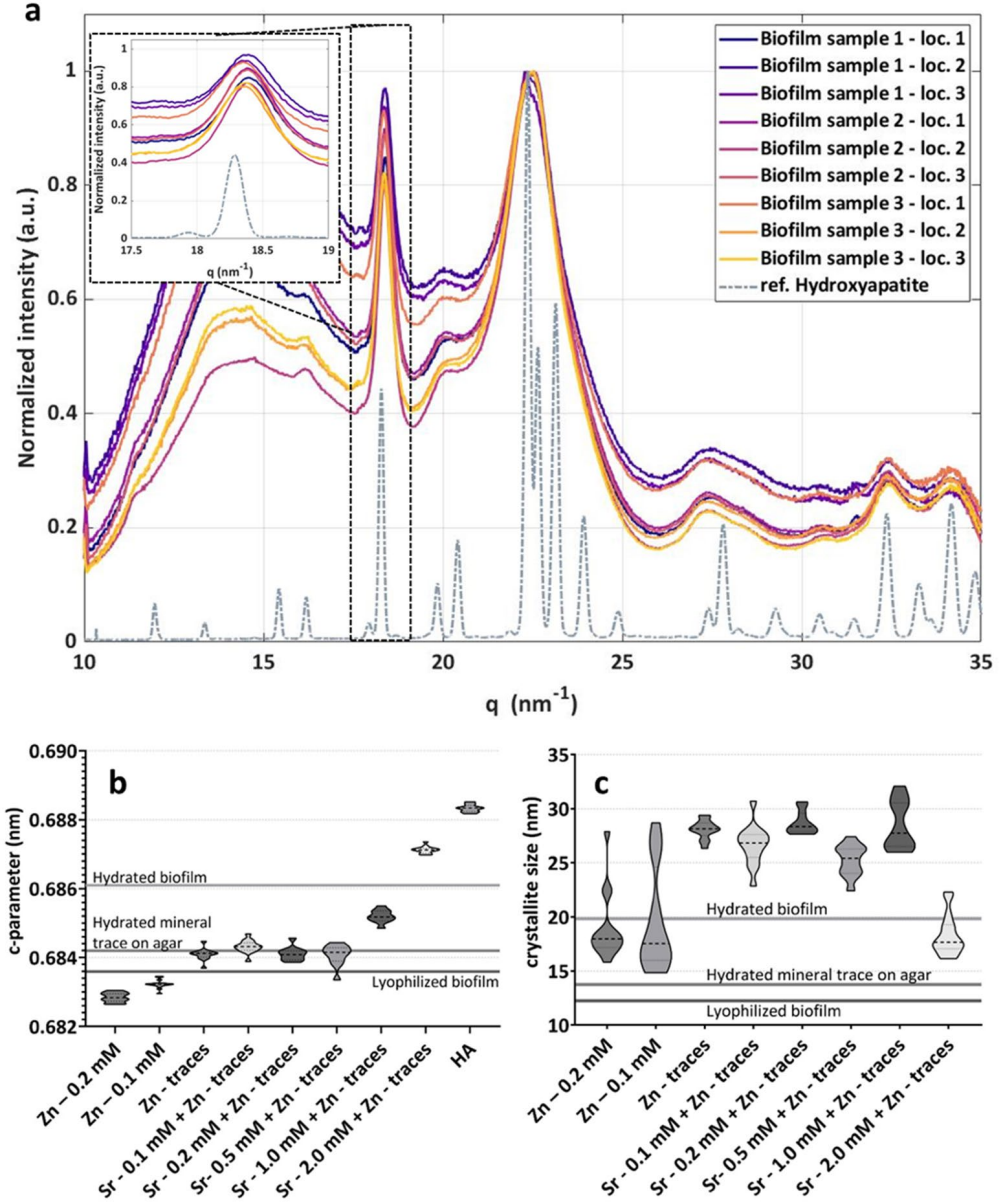
Wide angle X-ray scattering of biofilms and mineral precipitated in the abiotic system. **a** WAXS plot of 9 measurements of 3 lyophilized biofilms (Ca10P10) compared to standard hydroxyapatite powder with a zoom on the 002 peak. The intensity data are normalized subtracting the minimum intensity value and dividing the resulting values by the maximum intensity of the 112 hydroxyapatite peak. **b** Violin plots of c-parameter for the 002-peak of mineral precipitated with the abiotic system. **c** Violin plots of crystal size for the 002-peak of mineral precipitated with the abiotic system. In the violin plots, dashed lines indicate the median and the dotted lines the first and third quartiles of the distributions.

X-ray diffractograms of powder obtained from fixed and lyophilized mineralized biofilms were analyzed to identify and quantify potential crystal strains induced by such substitution. The data of 3 positions on three biofilms shows an average interplanar space along the crystal c axis (c-parameter) equal to 0.6836 nm (and a standard deviation of 0.006 nm, for *N* = 9) (Fig. 4a). The c-parameter for a standard hydroxyapatite powder measured with the same instrument is 0.6883 nm (standard deviation of 0.0001 nm, for *N* = 16), which indicates a lattice distortion in the biofilms of 0.5%. The distortion could be partially related to the freeze-drying post-processing but we showed in Fig. S1c that it does not affect the crystal phase^4^. However, the 002-peak of hydroxyapatite measured in biofilms closer to native state (fixed, but hydrated biofilm) indicates a smaller distortion compared to the standard with a c-parameter of 0.6861 nm (standard deviation of 0.0004 nm, for *N* = 12), which is compatible with zinc substitution in hydroxyapatite lattice^32^. To keep the conditions of the experiment similar to the native state, a biofilm was fixed and analyzed after removing excess nutritious agar and placed between two sheets of Kapton foil. In this way, we could differentiate the contribution on the crystal distortion related to the lyophilization and the one related to the growing environment (organic components and Zn presence in the nutritious medium) (Table 4). Analyzing the mineral traces observed in the agar below the biofilm revealed a c-parameter of 0.6842 nm (standard deviation of 0.001 nm, for *N* = 21), i.e. slightly lower compared to the hydroxyapatite formed in the biofilm and measured in the same conditions, but still larger than the hydroxyapatite formed in the biofilm and measured after lyophilization (Table 4; Fig. 4b). The Scherrer equation^33^ enables to estimate the crystallite particle dimensions perpendicular to the corresponding planes (c axis in case of 002 peak). Lyophilized Ca10P10 biofilms presented crystals with a dimension of about 12 nm along this axis (Table 4), whereas in hydrated conditions the size of the crystallites in biofilms was almost double (about 19 nm). While such difference suggests that the freeze-drying process may affect crystallite size, it may also result from different averaging scenario when lyophilized powder obtained from the whole biofilm are compared to a targeted mineralized region of the native hydrated biofilm.

To further check this theory of the crystal doping by Zn in a more systematic way, we designed a simple abiotic (i.e. bacteria-free) model where hydroxyapatite can be enzymatically precipitated from Ca ions and an organic source of P in the presence of different nominal concentrations of Zn^2+^ or Sr^2+^ ions. From MXRF measurements on these samples (see Supplementary Fig. S10) we have derived Ca/P and Zn/(Zn + Ca) ratios. Ratios are used here because the intensity values derived from the MXRF measurements do not follow a linear correlation with the concentrations of the elements (Ca, P, Zn). As the matrix and structure of the biofilms is complex and the thickness and density of the abiotic samples is not known, a quantification on these samples is not possible. However, the ratios allow to compare measurements on samples with a similar matrix measured with the same setup and measuring conditions. Using the ratios facilitates an approximate comparison of the intact biofilms, the abiotic samples and the pellets. We added information derived from measurements on HAp and biofilm pellets (Ca10P10 and control). The variation of the Ca/P ratio in the abiotic system samples is low and comparable with the values derived from HAp. The Zn/(Zn + Ca) follows the expected trend and is higher for the samples prepared with a higher nominal concentration of Zn. For all the samples, there is a nominal base level of Zn due to the buffer solution used for the ALP enzyme equal to 0.2 mM. Figure 4b displays the c-parameter (that is the size of the crystal unit along the c-axis, estimated through the 002-peak) and shows that higher Zn concentrations lead to a smaller lattice, whereas higher Sr concentrations lead to a larger crystal lattice (Table 4). The size of the entire crystallite along c-axis is shown in Fig. 4c. In this case, the presence of zinc appears to inhibit crystal growth, whereas the effect of increasing strontium concentration does not cause a clear effect on the crystallite size.

**Table 4:** c-parameter and crystal size of freeze-dried, hydrate biofilms and mineral precipitated in the abiotic system.

Sample	c-parameter	Lattice Distortion	Crystal size
Standard hydroxyapatite powder	0.6883 nm (SD = 0.0001 nm)	Reference	51.86 nm (SD = 5.06 nm)
Powder of fixed lyophilized mineralized biofilms (Ca10P10)	0.6836 nm (SD = 0.006 nm)	0.5%	12.25 nm (SD = 0.29 nm)
Fixed hydrated biofilms (Ca10P10)	0.6861 nm (SD = 0.0004 nm)	0.17%	19.83 nm (SD = 2.71 nm)
Mineral traces in the hydrated agar	0.6842 nm (SD = 0.001 nm)	0.45%	13.7450 nm (SD = 7.44 nm)
Vacuum-dried precipitate from abiotic system (Zn - traces)	0.6864 nm (SD = 0.0002 nm)	0.13%	28.30 nm (SD = 0.48 nm)

## Discussion

This study revealed that during *E. coli* biofilm mineralization mediated by ALP, the traces of Zn provided in the nutritive medium tend to accumulate together with calcium and phosphate in the mineralized regions. Using MXRF and CMXRF imaging with a spatial resolution in the range of tens of micrometers, the highly heterogeneous structure of the films could be visualized with different layers above and inside the agar substrate with a strong co-localization of Zn with Ca/P.

Currently, only a few studies in literature use XRF techniques on biofilm samples to reveal the lateral distribution of transition metals. For example, XRF in combination with XRD was used by Azulay et al.^34^ to study molecular heterogeneities within intact *Bacillus subtilis* biofilms. XRF revealed the distribution of Ca, Mn, Fe and Zn across the biofilm. Yang et al. used XRF to investigate the elemental distribution of Se and other elements in multispecies biofilms^35^. While the XRF technique is not well established in the fields of biofilm imaging, our study emphasizes its usefulness to reveal the elemental distribution in intact biofilms in two to three dimensions on a macroscopic length-scale. In contrast to SEM-EDX, the sensitivity of the XRF technique for higher-Z elements such as Zn makes it suitable for ppm imaging. As the sample preparation is simple and the measurement can be conducted in a non-destructive manner leaving the biofilm in a wet, intact state, high-resolution techniques can be performed afterwards on specific selected areas of the rather large samples (> 1 cm).

The process of X-ray fluorescence production is a non-linear process which is well described^36^ with all relevant cross sections available from data bases^37^. Therefore, in a best-case scenario, intensities can be converted to absolute concentrations of all elements in a sample. When using hard X-rays and commercially available instrumentation, light elements such as carbon, oxygen or nitrogen cannot be detected, which necessitates a good knowledge of this so-called dark matrix. Additionally, if the sample investigated is not infinitely thick, i.e. in the range of centimeters for biological matrices, the density and thickness must also be known. Both factors limit the possibility for absolute quantification.

We present here the analysis of homogeneous pressed pellets of each of the biofilm components, where density and thickness are well known (Table 3). In this case, because the dark matrix is derived through a best guess, the results are semi-quantitative and the uncertainties are high. In the case of the native biofilms, though, the thickness and density change laterally and into the depth, thus, rendering a quantification not feasible. Nonetheless, a relative comparison of intensities can be conducted for elements with similar sensitivities and information depths (Figs. 1, 2 and 3). We can compare the ratio of the Ca and P K MXRF intensities between different biofilms, because the radiation originates from surface-near regions. The ratio does not change significantly with time and is roughly in the range of a HAp reference measurement (see Supplementary Fig. S6). As no absolute quantification was performed, the Ca/P ratios cannot be compared with literature values (Ca/*P* = 1.67, ideal stoichiometry of HAp). Therefore the different sensitivities of the setup for Ca and P need to be considered. Nevertheless, measurements acquired using the same setup, same measurement parameters and similar matrix can be compared relative to each other. While P and Ca originate from a surface-near layer, Zn on the other hand can be detected from > 300 μm depths, thus, the MXRF intensity is the sum of all layers visible in Fig. 3a, including the ring-like structure in the agar (see Supplementary Fig. S8). As the amount of Zn present in all components and the measured biofilm pellets is below 200 ppm, its mass fraction is 2 to 3 orders of magnitude smaller than the mass fractions of P and Ca. Therefore, even if all Zn were substituting Ca in the HAp crystals, the ratio of Ca to P would not change significantly.

Nonetheless, the co-localization of Zn and Ca/P demonstrates that Zn is either in or around the mineralized crystals, which is coherent with its high affinity for the ALP enzyme involved in *E. coli* biofilm mineralization^4,19^. Moreover, the hydroxyapatite formed in the biofilms was found to have a distorted crystal lattice, with a c-parameter shorter than in reference hydroxyapatite (Fig. 4a). For the hydroxyapatite in bone, a residual mineral strain of 0.15% is related to a pre-stress of the order of 100 MPa^38^. However, mineral in biofilms forms in a highly heterogeneous environment. For this reason, it is not trivial to relate the measured distortion to a pre-stress. Such distortion also occurred upon enzymatic precipitation of hydroxyapatite in an abiotic model system without bacteria nor extracellular matrix. Since traces of Zn are required in the buffer to activate the enzyme ALP, we cannot test if the enzyme alone affects crystal distortion in this system, nor rule out this possibility. Yet, hydroxyapatite lattice distortion increased with the concentration of Zn at constant ALP concentration, suggesting that part of the Zn present in the biofilm environment may have substituted Ca atoms in the hydroxyapatite crystal. These different scenarios are neither contradictory nor exclusive.

To assess how much of Zn is incorporated in the HAp lattice versus bound to the biofilm extracellular matrix, it may be beneficial to compare the elemental composition (XRF) and crystallography (WAXS) of mineralized biofilm powder and of purified biofilm minerals. Considering the potential artifacts induced by the use of chemical and/or enzymatic treatments to remove the organic phase of the biofilm, such approach should first be validated using minerals enzymatically precipitated in the abiotic (bacteria-free) system to confirm that the chemical treatment itself does not affect mineral crystallography.

The Ca/P ratios derived from MXRF measurements of the abiotic system samples prepared with 0.2 mM and 0.1 mM zinc do not change significantly and are in the same order of magnitude as the values derived from a HAp pellet (see Supplementary Fig. S10). While these ratios of the abiotic system cannot be directly compared to the measurements on biofilms (see Supplementary Fig. S6) due to different conditions during the measurement, the measurements on HAp give a hint that the values are in the same order of magnitude with the ratios derived from the biofilm samples. The same holds true for the Zn/(Zn + Ca) ratios of the abiotic system prepared with 0.2 mM and 0.1 mM zinc, while this ratio for the samples with only Zn traces is much lower.

Zinc is a ubiquitous element essential to bacteria metabolism^6^, but also a widely-used antibacterial agent^15,16^. Bacteria thus developed various strategies to tightly maintain Zn homeostasis^39^. In *E. coli*, the target intracellular concentration of Zn is about 0.2 mM, however, the quasi-totality is bound to Zn(II)-binding molecules in their cytoplasm^40^. In conditions allowing for enzymatic mineralization, Zn-doping of hydroxyapatite could represent an additional / alternative way for *E. coli* to manage Zn excess. As a consequence of Ca substitution by the larger element Zn, the crystal lattice of hydroxyapatite is distorted. Although not unanimous on the type of distortion^41^, a majority of studies characterizing zinc-doped hydroxyapatite also showed a decrease of the c-parameter as Zn concentration increased in the mineralization environment^42–44^ (Fig. 4c). Depending on the study, the values of c-parameter reported for hydroxyapatite and Zn-doped hydroxyapatite in such studies appear to be slightly higher than ours (Table 4), but the trend is similar^42,43^. This could be explained by differences in samples preparation, e.g., in drying process. Together with a decrease of the hydroxyapatite c-parameter, a rather old work also reported a decrease of the crystal size with increasing presence of Zn and suggested an inhibiting effect of zinc on hydroxyapatite crystallization^44^. This hypothesis is further supported by results obtained in the abiotic model (Fig. 4c), showing that the crystal size of hydroxyapatite issued from enzymatic precipitation in the presence of Zn (even at very small concentrations) is significantly smaller than pure hydroxyapatite powder. This study also shows that HAp crystal size decreases with Zn content^45^.

Antimicrobial properties of Zn-doped HAp are of particular interest for tissue engineering applications. Zn ion release quantification around Zn-doped HAp samples and measurements of the subsequent growth inhibition area showed promising results against *E. coli* and *S. aureus*^32^. It was shown that the toxicity of ZnHA on *E. coli* is somewhat relative at low Zn concentrations but other species may be more sensitive (*S. aureus* for example)^21,46^. To investigate the interplay between Zn and bacteria during biofilm formation and mineralization in clinical settings, the methodology established in the present work should be applied to experimental systems of higher contextual relevance. For example, bacterial strains such as *Proteus mirabilis* could be involved in studies about kidney stones, whereas oral bacteria such as Streptococcal strains or even saliva samples containing patient’s microbiome could be considered to study dental calculus. In the case of oral biofilms, the approach should be applied to stiffer substrates that more appropriate than agar to mimic dental surfaces, as in the artificial mouth model presented recently^47^.

Zn was also shown to influence calcium carbonate biomineralization of Halomonas halophila^48^. In this case, it seems that bacteria accumulate minerals on their surface and that Zn concentration in the surroundings affects biomineralization (from calcite to other polymorphs of CaCO_3_). While Zn concentration within the bacteria remains in acceptable range for the organisms, it is not clear if extracellular Zn is incorporated in the mineral crystals or only in the organic part of the extracellular matrix^48^. Better understanding of Zn incorporation in biofilms could thus lead to optimization of biofilm applications such as bioremoval of zinc and manganese^49^.

Overall, this work highlights the potential of combining X-ray fluorescence and X-ray scattering techniques to address the understudied question of Zn management in mineralized biofilms. It reveals that the traces of Zn from the growth medium accumulate in the mineralized regions of the biofilms, and suggests that part of this Zn substitutes calcium within the mineral crystals, yielding Zn-doped hydroxyapatite. Considering the importance and ubiquitous role of trace elements in biofilm formation, such knowledge and methodological approach will have promising implications in the fields of medicine, geomicrobiology and bioremediation.

## Supplementary Information

Below is the link to the electronic supplementary material.

Supplementary Material 1

## Data Availability

The datasets that support the findings of the current study are available from the corresponding author on reasonable request.
