# Supplementary material for: Zinc and hydroxyapatite co-localize during in vitro E. coli biofilms mineralization
Source: Sci Rep. 2026 Jan 14;16:2059. doi: 10.1038/s41598-025-33942-3 (PMC12808131; doi:10.1038/s41598-025-33942-3)
Supplement: Supplementary file 1 — Supplementary Material 1 [file 41598_2025_33942_MOESM1_ESM.docx]

Supplementary Information for

Zinc and hydroxyapatite co-localize during in vitro E. coli biofilms mineralization

Leona J. Bauer^1,3*^, Laura Zorzetto^2*^, Ernesto Scoppola^2^, Yannick Wagener^1^, Ioanna Mantouvalou^1,3†^ and Cécile M. Bidan^2†^

^1^ Institute for Physics and Astronomy, Technische Universität Berlin, Hardenbergstrasse 36, 10623 Berlin, Germany.

^2^ Max Planck Institute of Colloids and Interfaces, Department of Biomaterials, 14476 Potsdam, Germany.

^3^ SyncLab, Helmholtz-Zentrum Berlin für Materialien und Energie, Albert-Einstein-Str. 15, 12489 Berlin, Germany.

^*^ equal contributions

^†^ corresponding authors

Corresponding authors: [Cecile.Bidan@mpikg.mpg.de](mailto:Cecile.Bidan@mpikg.mpg.de), ioanna.mantouvalou@helmholtz-berlin.de

| 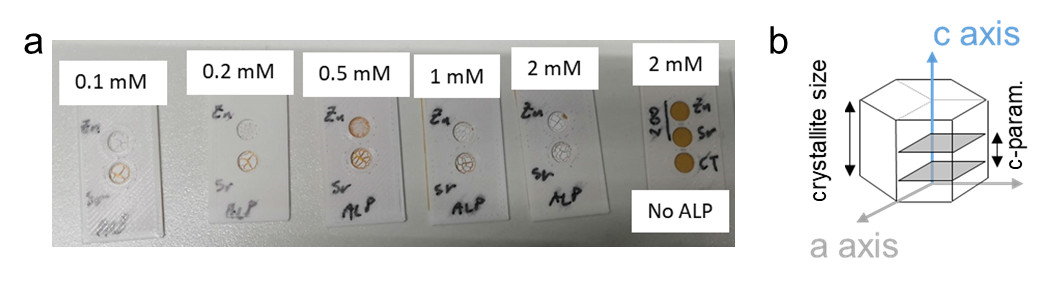 |
| --- |
| 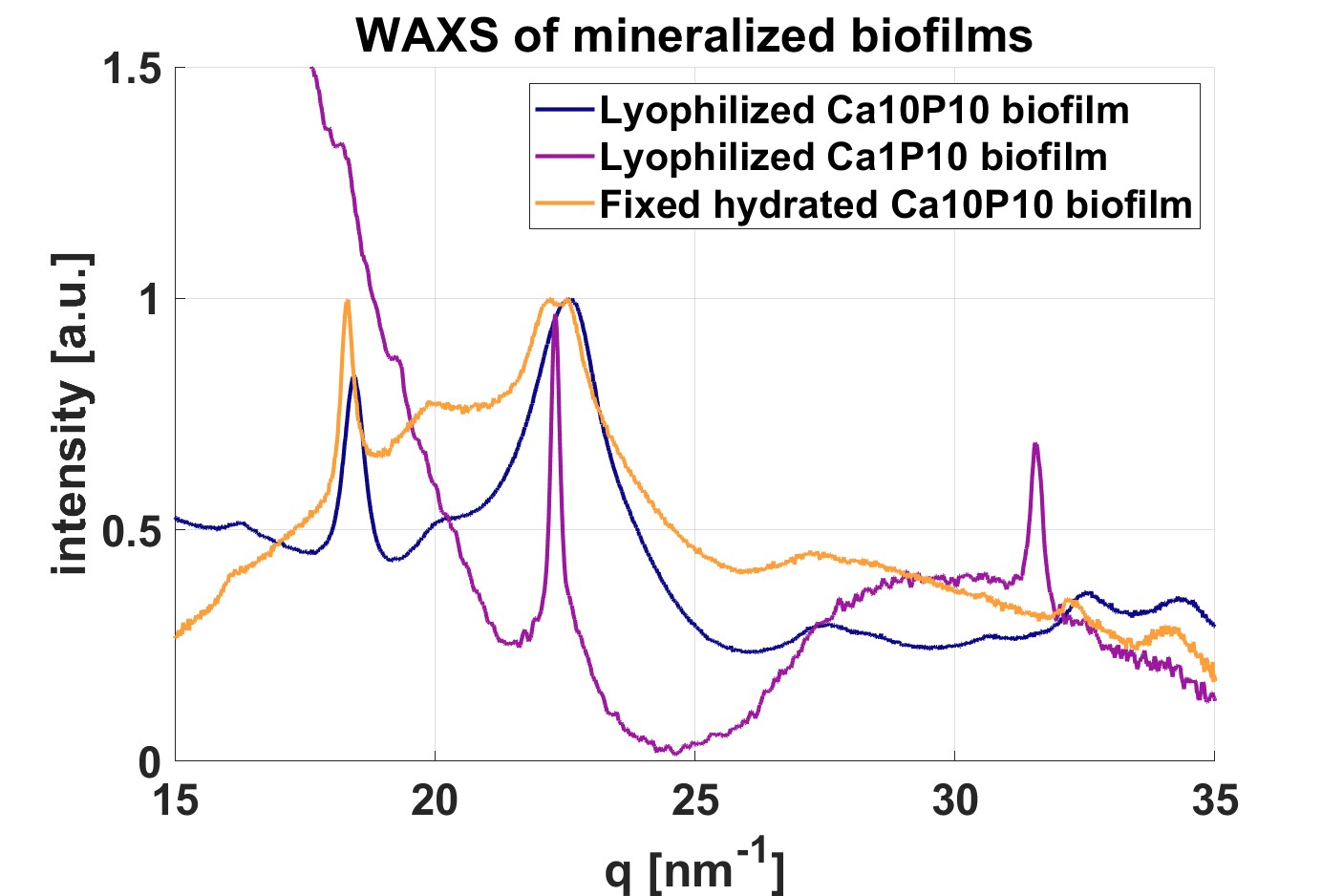c |

Figure S1: a Sample holders containing enzymatically precipitated hydroxyapatite with different ZnCl_2_ and SrCl_2_ concentrations. Control samples with No ALP were also measured, although no precipitate was visible. b scheme illustrating the definition of crystallite size and c-parameter within a mineral crystal. c X-ray scattering patterns of lyophilized Ca1P10 and Ca10P10 biofilms and of fixed hydrated Ca10P10 biofilms. The intensity data are normalized subtracting the minimum intensity value and dividing the resulting values by the maximum intensity of the 112 hydroxyapatite peak.


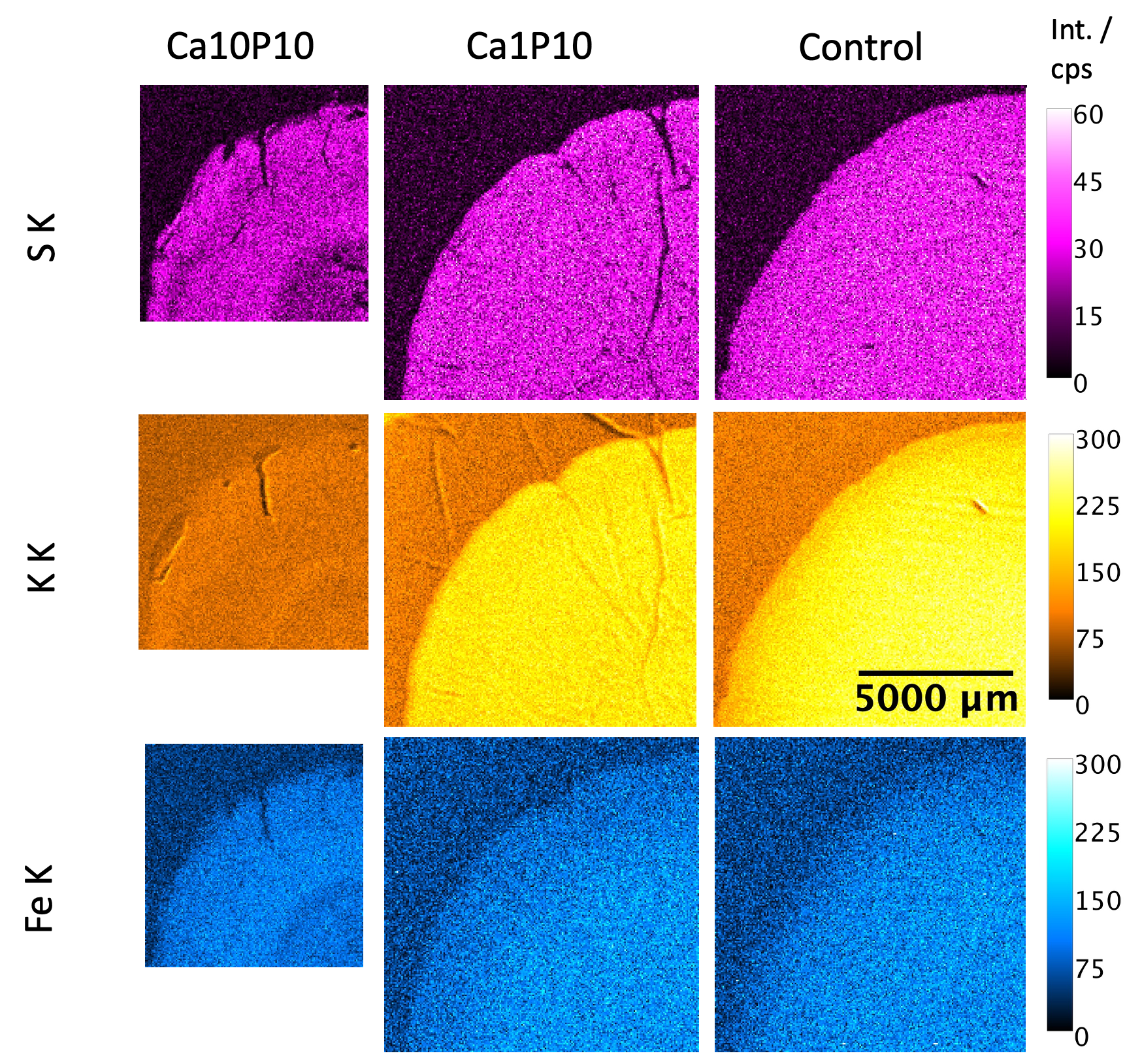


Figure S2: Additional elemental distributions obtained from MXRF measurements on three biofilms grown for 10 days under different mineralization conditions (Ca10P10, Ca1P10, Control). The step size is 50 µm and the measurement times are 1.75 s, 0.85 s, 0.85 s (25 ms, 70 cycles and 35 cycles). The color scales show the X-ray fluorescence net peak intensities in counts per second (cps). The length scale is the same for all three measurements.


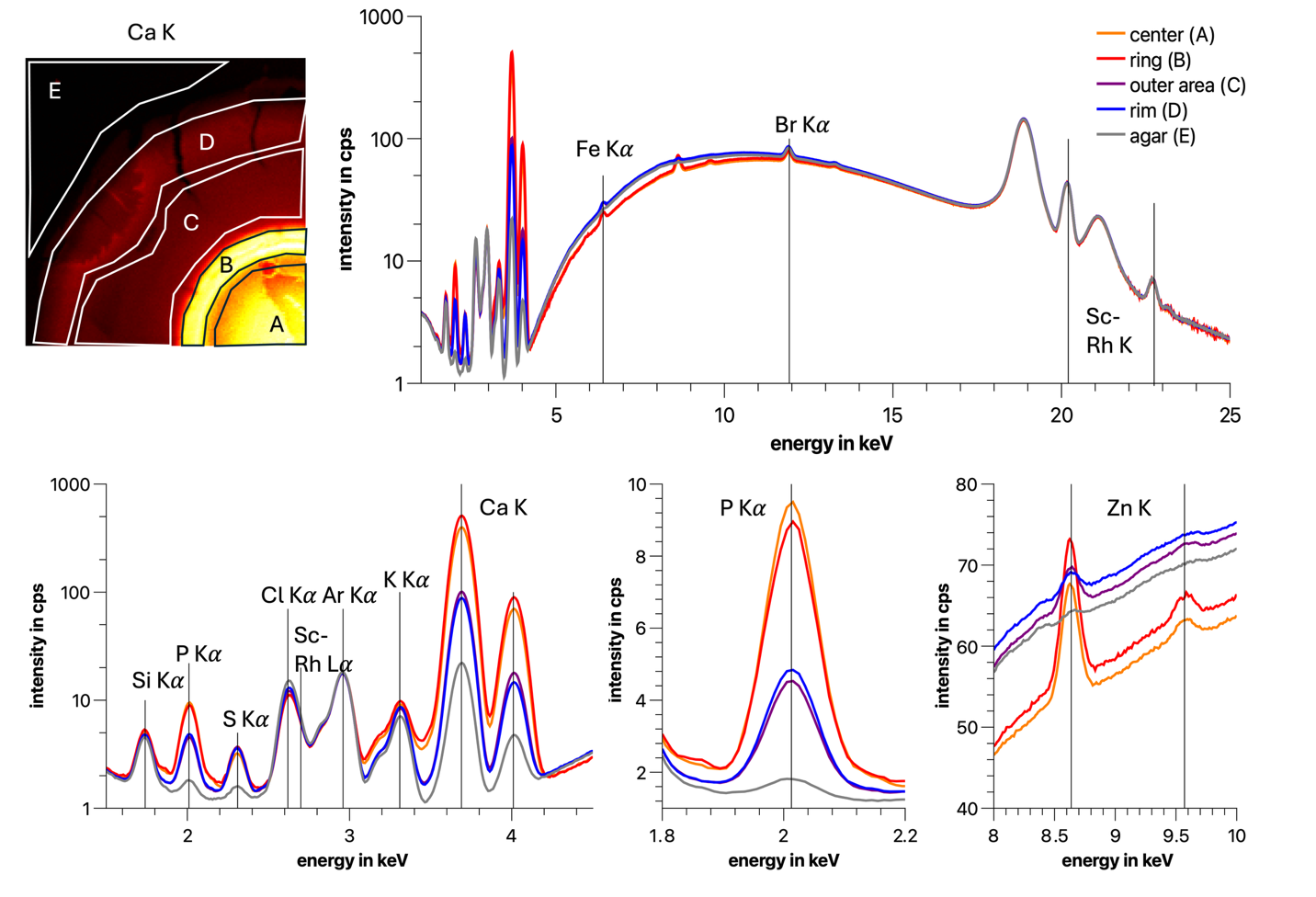


**Figure S3:** Averaged spectra of the marked areas on the measurement on a Ca10P10 biofilm. The full spectra are presented, along with three zoom-ins highlighting the peaks corresponding to Ca, P and Zn.


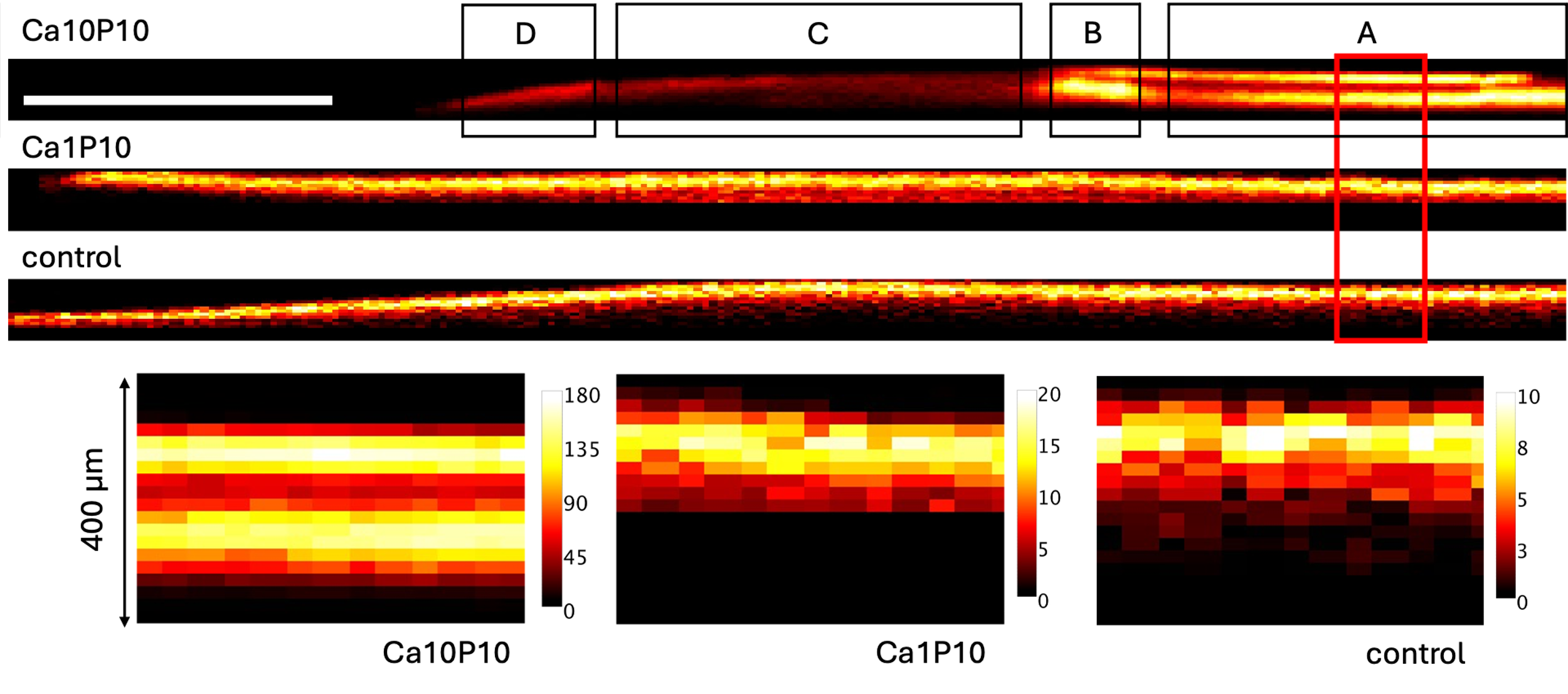


**Figure S4:** Virtual slices into the depth for all biofilms shown in Fig. 1 grown for 10 days under different mineralizing conditions (Ca10P10, Ca1P10, control). The scalebar is 2 mm and the color bars show intensities in cps. Areas A, B, C, and D indicate the approximate regions used for the averaged spectra in Fig. S3.


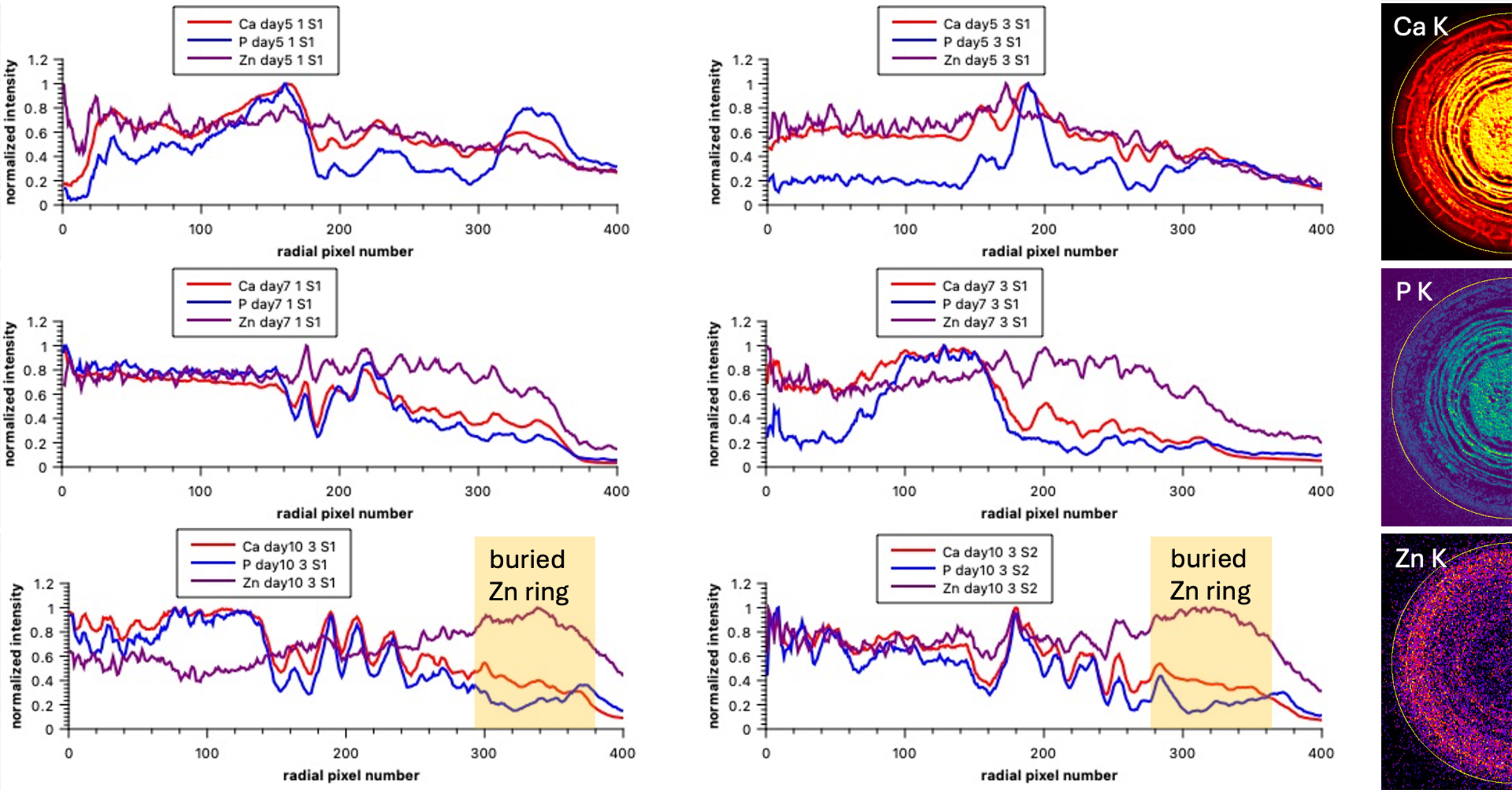


**Figure S5:** Radial profiles derived from the MXRF measurements shown in Fig. 2. The radial profiles were calculated using the “Radial profile extended” plugin in ImageJ (by Philippe Carl). A yellow semicircle in the elemental profiles on the right side shows the used angle and radial range. The higher Zn intensity towards the rim of the biofilm in the day ten samples can be explained by the Zn accumulation in the Agar, see Figure 3.


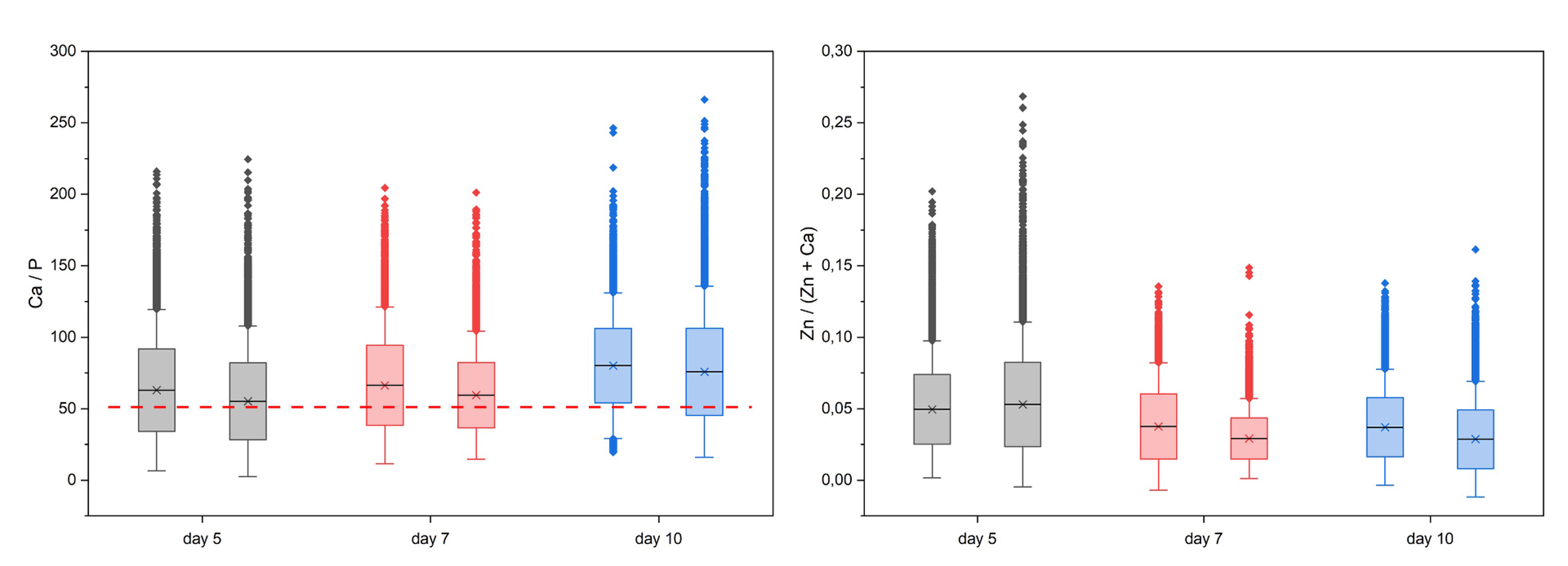


Figure S6: Ca/P (left) and Zn/(Zn+Ca) (right) ratio of the biofilms grown for 5, 7 and 10 days. The boxplot shows the mean value, standard deviation and outliers of the ratios derived from the net peak intensities derived from 2D maps on the biofilms grown for 5, 7 and 10 days (Figure 2). The values cannot be compared with values reported in literature, because the setup specific sensitivity, which differs for Ca and P plays a role. Ratios can only be compared, when measured with the same setup and same measurement conditions. The red dotted line shows the Ca/P ratio derived from the measurement on a HAp pellet using the same setup.


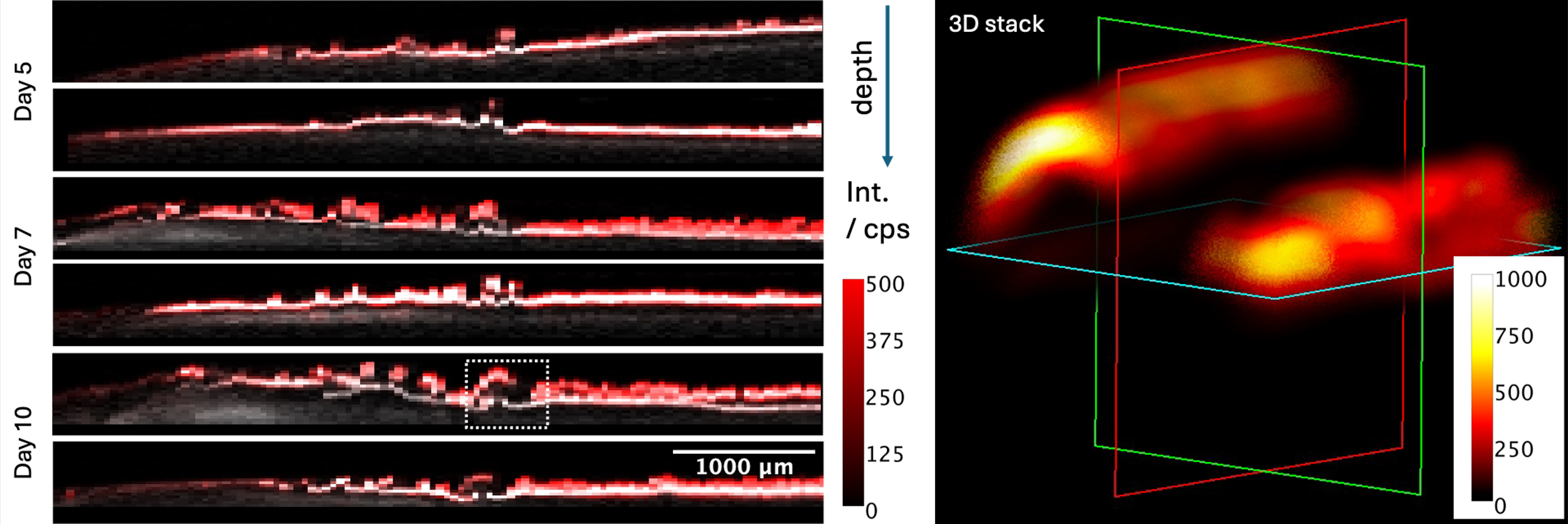


Figure S7: Left: Ca K distributions of virtual slices into the depth for all biofilms in Fig. 3 along the white dotted line measured by CMXRF with a step size of 20 µm into the depth, 50 µm lateral step size and a measurement time of 51 s. Ca K distribution is shown in red as an overlay on the Zn K distribution in gray. Right: 3D Ca K distribution of a detail of a biofilm grown for 10 days (marked with the white rectangle, 20 µm x 20 µm x 20 µm step size, 18.5 s measuring time.


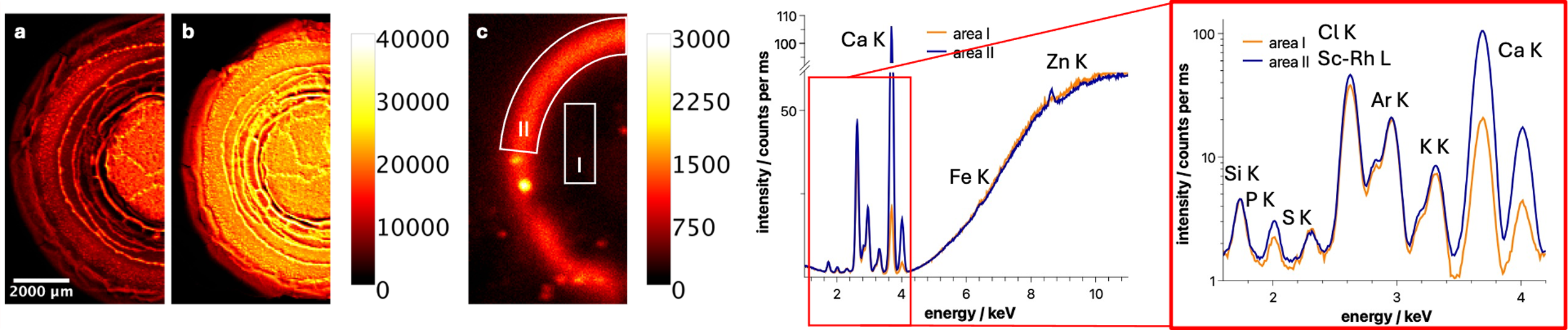


Figure S8: (Left) Ca Kα distribution of a MXRF measurement on a a biofilm on agar substrate, b biofilm removed from agar substrate, c agar substrate after removal of the biofilm. Mineral accumulation also happens in the agar. The step size was 50 µm and the measuring time 200 ms. (Right) Summed spectra with respective fluorescence lines of areas I and II marked in white in c. A rhodium (Rh) tube is used for excitation, thus, the characteristic Rh scattering peaks (elastic and inelastic, Sc-Rh L) are seen. Argon (Ar) fluorescence is emitted from the air.

Table S1: Assumptions for the dark matrix for quantification of pellets

|  | information | dark matrix approximation |
| --- | --- | --- |
| **agar** | 70 % agarose: C_12_H_18_O_9_  30 % agaropectin:  cellulose: C_12_H_20_O_10_ | 99.25 % (C_12_H_18_0_9_) |
| **yeast**  **on C-tape** | >8 % N,  yeast | 52 % (CH_158_N_28_O_30_)  40 % (C_6_H_12_O_6_)  (proteins + carbs) |
| **tryptone**  **on C-tape** | >10 % N,  tryptophan rich: C_11_H_12_N_2_O_2_ | 94.5 % (C_11_H_12_N_2_O_2_) |
| **E. coli**  **on C-tape** | E. coli  70 % H20, 15 % proteins, 13 % (DNA, RNA, carbs, small molecules), 2 % phospholipids | 95 % (CH_158_N_28_O_30_)  (proteins) |
| **biofilm**  **Ca10P10**  **on C-tape** | HA: Ca_5_(PO_4_)_3_OH  + 1.8 % E. coli  + 1 % tryptone  + 0.5 % yeast  + CaCl + Sodium ß-glycerophosphate  40 % mineral, 60 % bacteria | 87 % (C_12_H_170_N_30_O_32_)  (tryptone + E.coli) |
| **biofilm**  **control** | + 1.8 % E. coli  + 1 % tryptone  + 0.5 % yeast | 95 % (C_12_H_170_N_30_O_32_)  (tryptone + E.coli) |


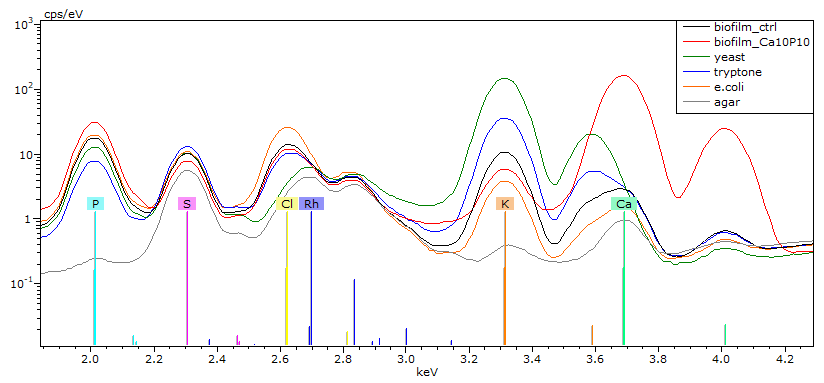


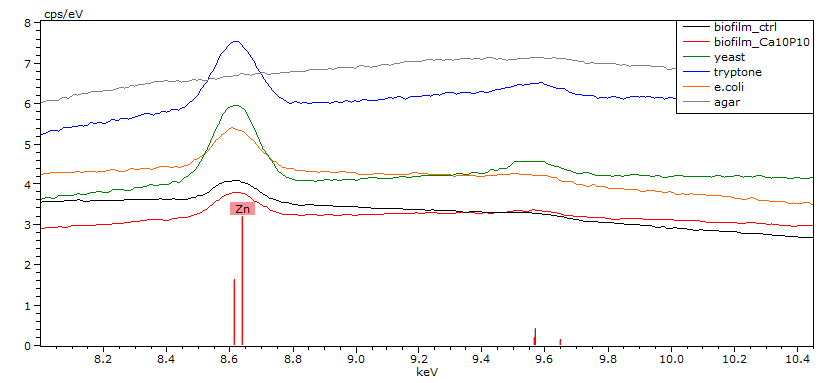


**Figure S9:** Sum spectra with respective fluorescence lines obtained on pressed pellets from the powders. A Rh tube is used for excitation, thus, the characteristic Rh scattering peaks (elastic and inelastic) are seen. Top: main elements, bottom: Zn K fluorescence lines.


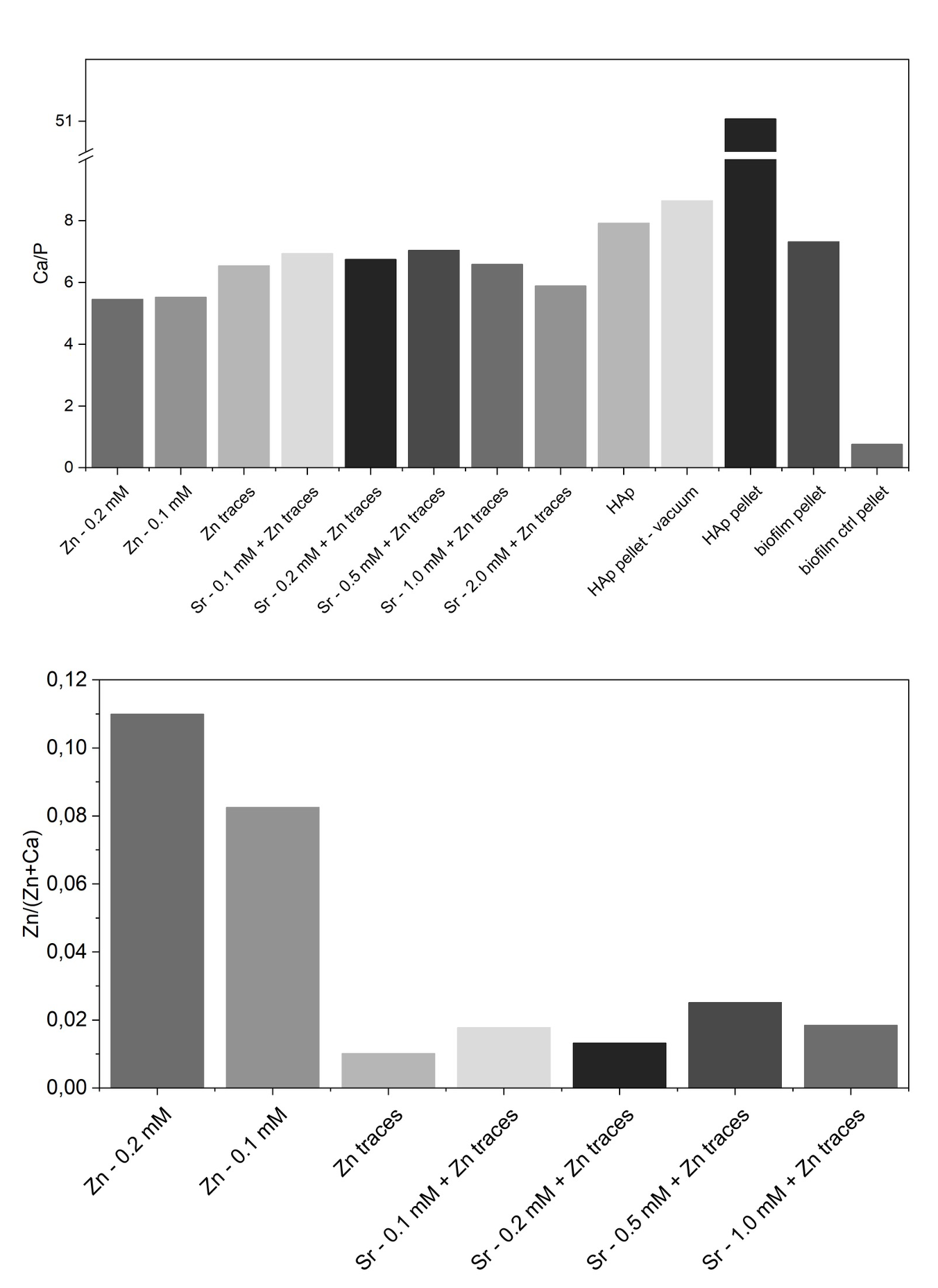


**Figure S10:** Ca/P (top) and Zn/(Zn+Ca) (bottom) ratios of the abiotic samples, HAp and biofilm pellets derived from MXRF measurements. The samples were measured in vacuum, which prevents a direct comparison to the ratios shown in Figure S3. Due to that we added the Ca/P ratios of two measurements on the HAp pellet – one measured in vacuum (20 mbar) and one measured under ambient pressure. This allows to assume a shift of the values derived from the abiotic system to higher values. For the Zn/(Zn+Ca) ratios one would expect a shift to lower values, due to the better sensitivity for Ca K under 20 mbar, while Zn K is not so much affected.
